# Rspo1-activated signalling molecules are sufficient to induce ovarian differentiation in XY medaka (*Oryzias latipes*)

**DOI:** 10.1038/srep19543

**Published:** 2016-01-19

**Authors:** Linyan Zhou, Tapas Charkraborty, Qian Zhou, Sipra Mohapatra, Yoshitaka Nagahama, Yueguang Zhang

**Affiliations:** 1 Key Laboratory of Freshwater Fish Reproduction and Development (Ministry of Education), Key Laboratory of Aquatic Science of Chongqing, School of Life Science, Southwest University, Chongqing 400715, P.R. China; 2 SORST, Japan Science Technology Corporation, Kawaguchi, Saitama 332-0012, Japan; 3 South Ehime Fisheries Research Center, Ehime University, Ainan, Ehime, 798-4206, Japan; 4 Graduate School of Life and Environmental Sciences, University of Tsukuba, 305-8577, Ibaraki, Japan; 5 Institution for Collaborative Relations, Ehime University, 790-8577, Matsuyama, Japan

## Abstract

In contrast to our understanding of testicular differentiation, ovarian differentiation is less well understood in vertebrates. In mammals, R-spondin1 (Rspo1), an activator of Wnt/β-catenin signaling pathway, is located upstream of the female sex determination pathway. However, the functions of Rspo1 in ovarian differentiation remain unclear in non-mammalian species. In order to elucidate the detailed functions of Rspo/Wnt signaling pathway in fish sex determination/differentiation, the ectopic expression of the *Rspo1* gene was performed in XY medaka (*Oryzias latipes*). The results obtained demonstrated that the gain of *Rspo1* function induced femininity in XY fish. The overexpression of *Rspo1* enhanced *Wnt4b* and *β-catenin* transcription, and completely suppressed the expression of male-biased genes (*Dmy, Gsdf, Sox9a2* and *Dmrt1*) as well as testicular differentiation. Gonadal reprograming of *Rspo1*-over-expressed-XY (*Rspo1*-OV-XY) fish, induced the production of female-biased genes (*Cyp19a1a* and *Foxl2)*, estradiol-17β production and further female type secondary sexuality. Moreover, *Rspo1*-OV-XY females were fertile and produced successive generations. Promoter analyses showed that Rspo1 transcription was directly regulated by DM domain genes (*Dmy*, the sex-determining gene, and *Dmrt1*) and remained unresponsive to Foxl2. Taken together, our results strongly suggest that Rspo1 is sufficient to activate ovarian development and plays a decisive role in the ovarian differentiation in medaka.

Sexual differentiation represents a tug of war between male-dominated and female-activated genes in mammals^1^,^2^. In XY gonads, transiently activated *SRY*/*Sry* gene expression in the supporting cell lineage has been shown to initiate the male differentiation pathway by up-regulating *Sox9* and *Fgf9* expression^3^,^4^,^5^,^6^. On the other hand, although several candidate genes are known to be important in female sex differentiation, the master regulator/regulatory pathway has not yet been identified for XX embryos. Previous studies revealed that a Foxl2-leading pathway and Rspo1-activating signalling pathway act independently and complementary to each other, in order to promote ovarian development^1^,^7^,^8^,^9^,^10^,^11^,^12^,^13^.

In mammalian species, *RSPO1/Rspo1* is involved in ovarian determination and differentiation, by synergizing with specific Wnt ligands to stabilize intracellular β-catenin^1^,^14^. Murine *Rspo1*, specifically up regulated in the XX gonad at E11.5, is necessary for gonadal somatic and germ cell differentiation^10^,^15^. In XX mouse gonads, the loss of Rspo1 induces ectopic steroidogenic precursors, endothelial cell migration, formation of a coelomic vessel, Sox9 expression in supporting cells lineages, and formation of ovotestis^16^,^17^. RSPO1 is specifically expressed in the foetal ovary between 6 and 9 weeks after conception^18^, and a single nucleotide insertion in the *RSPO1* gene results in sex reversal and testicular development in XX humans^19^,^20^. A previous study revealed that the duplication of chromosome 1p, containing *Rspo1* and *Wnt4* loci, resulted in male-to-female sex reversal in humans^18^. The stabilization of β-catenin, the downstream gene of *Rspo1*, in the XY gonad successfully induces sex reversal, suggesting that elevations in *Wnt4* and *Rspo1* or their downstream signals are sufficient to override testicular development^9^. Therefore, the Rspo1/Wnt/β-catenin pathway might be instrumental in repressing the Sry-activated male pathway, which, in turn, activates ovarian determination and differentiation in XY mice. Recent findings demonstrated that *Rspo1* displays a conserved, female-specific increase in expression in non-mammalian vertebrates, which highlights its conserved role in ovarian development^21^. Avian *Rspo1* is predominantly expressed in the ovaries, and was found to be down regulated in hormone-induced sex reversed ZW embryos^21^,^22^. In medaka, up-regulated levels of *Rspo1* were noticed in the gonadal primordium during the sex determination period^23^. *Rspo1* expression was detected in the somatic cells of zebrafish from 30 days after fertilization (daf) to 150 daf, as well as in germ cells between 30 and 60 daf in the ovary^24^. These findings emphasize the importance of *Rspo1* in non-mammalian vertebrate sex differentiation. However, the function of *Rspo1* has not yet been corroborated in teleostean species.

Medaka (*Oryzias latipes*) is a much appreciated model for sex determination and differentiation studies^25^,^26^,^27^,^28^,^29^,^30^, owing to its XX-XY heterogamety, thorough embryology, *Dmy/Dmrt1b*-based genetic sex determination, and distinct sex-specific secondary sex characteristics. We have previously demonstrated that over-expression of *Dmy* in XX medaka favours male specific genes expression and induces female to male sex reversal^29^, while XY *Dmrt1* (the downstream gene of *Dmy*) mutant medaka shows female specific genes expressions and further ovarian development^30^. This suggests that, both *Dmy* and *Dmrt1* are crucial to maintain the balance between the male- and female-dominated gene transcriptions, which eventually determines the gonadal sexuality in medaka^29^,^30^. Recently, we also recorded a female-biased and oestrogen-responsive *Rspo1* transcription during the sex determination period in medaka^23^. Previous *in vivo* ChIP analysis demonstrated that Dmy/Dmrt1b are adjoined to the *Rspo1* gene promoter thereby suggesting the interaction between Dmy/Dmrt1b and Rspo1 in medaka^31^. Therefore, in order to assess the importance of Rspo1 in the induction of femininity, in the present study, *Rspo1* was overexpressed in XY fish and sexual development was chronologically investigated. The relationship between the master sex-determining gene *Dmy* and *Rspo1* was also examined to substantiate the sex differentiation mechanism in medaka. Our results reveal that *Rspo1* is sufficient to activate female sex determination/differentiation in medaka. To the best of our knowledge, this is the first study to functionally characterize *Rspo1* in a non-mammalian vertebrate.

## Results

### The *Rspo1* transgene and GFP expression

**Figure 1:**
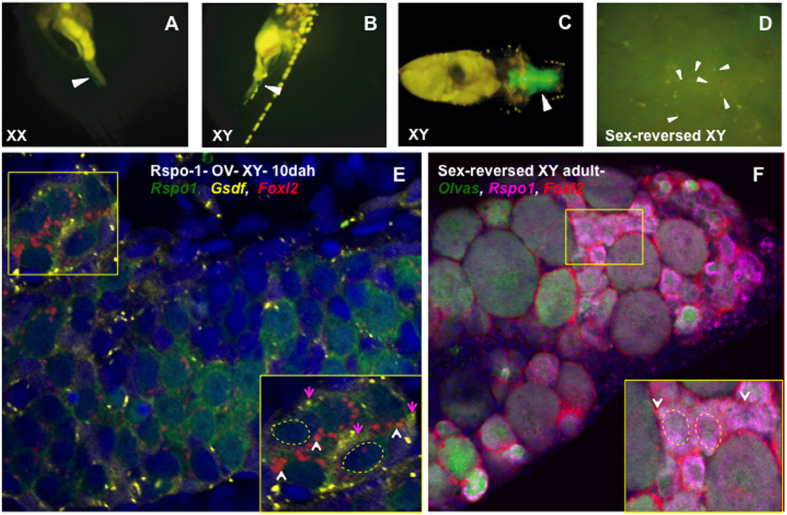
Localization of Rspo1 transgene in XY medaka embryos and adults. Rspo1-GFP expression (arrowheads) in gonads of *Rspo1* overexpressed XX (**A**) and XY (**B**) at 5 dah. Strong *Rspo1*-GFP expression is also seen in the brain (**C**) at 5 dah. In adults, GFP fluorescence (marked with white arrow head) is found in various gonadal cells (**D**). Photographs were taken from F_1_ generation embryos and F_0_ adults. Multicolor- fluorescent *in situ* hybridization FISH shows that *Rspo1* (green) expresses in germ cells and germ cell surrounding cells of 10 dah *Rspo1*-OV-XY fish in which either *Foxl2* (red) or *Gsdf* (yellow) expressions are evident (**E**). The representative *Foxl2-Gsdf* and *Rspo1-Gsdf* expressing somatic cells are respectively marked with white and pink arrowheads in the inset. The yellow dotted line represents candidate germ cells. Similar analysis shows that, *Rspo1* (magenta) mRNAs are congregated in the young oocytes, early germ cells and early germ cell surrounding cells at 60 dah (**F**). The representative *Foxl2-Rspo1* expressing early germ cell surrounding cells are marked with white arrowhead in the inset.

In order to determine the importance of Rspo1 in ovarian development, we constructed pIRES-hrGFP1a-Rspo1 plasmid by fusing *Rspo1* ORF with hrGFP1a, in order to drive the hCMV promoter dependent Rspo1-GFP fused protein expression in XY medaka (Fig. S1A). Subsequently, the linearized pIRES-hrGFP1a-Rspo1 plasmid was injected into XY medaka embryos at the one/two-cell stage and individually grew them on 24-well plates (1 embryo/ well) at 26 °C. The microinjected embryos were periodically screened for GFP expression, and the GFP-positive embryos were grown until adulthood. Genomic PCR was performed using a transgene-specific primer pair to confirm the genome integration of the *Rspo1* transgene (Fig. S1B). Live fluorescent microscopy depicted strong GFP signals in cells surrounding the germ cells in the XY sex-reversed ovary (Fig. 1A,D). Strong GFP expression was detected in the gonads and brains of 5 days after hatching (dah) embryos in the F1 generation (Fig. 1B,C). To further check the tissue specificity of Rspo1 transgene, we analysed the expression level of *Rspo1* in brain, gonad and the remaining body (RB) in *Rspo1*-OV-XY (*Rspo1*- overexpressed XY) fish by real-time PCR. The results showed that *Rspo1* gene expression was significantly higher in gonad and brain than the other tissues (Fig. S1C). In gonads, the GFP expression was scattered in germ cells and germ cell surrounding cells (Fig. 1E,F). Furthermore, multicolour-fluorescence *in situ hybridization* (*FISH*) was performed using different stages of Rspo1-OV-XY fish gonads to identify the cell type in which GFP expressions were prevalent in transgenic gonads. Abundant expression of *Rspo1, Foxl2* (female-biased genes) and *Gsdf* (male dominated) gene was observed in *Rspo1*-OV-XY fish at 10 dah (Fig. 1E). Meanwhile, co-localization between *Rspo1* and *Gsdf, Foxl2* and *Gsdf* were also found in germ cell surrounding somatic cells (Fig. 1E inset). Similarly, co-localization of *Rspo1* and *Vasa* was detected in the cytoplasm of young oocytes (Fig. 1F). Meanwhile, partial overlap of *Rspo1* and *Foxl2* gene expression in the germ cell surrounding cells was also observed (Fig. 1F, inset).

### Ectopic *Rspo1* expression induced ovarian development in XY fish

**Figure 2:**
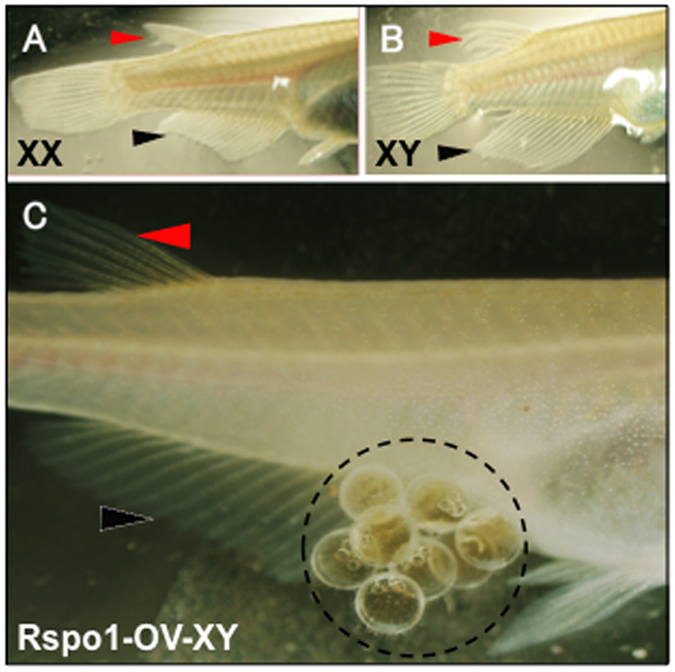
Fin structures and fertility of *Rspo1*-OV-XY medaka. XX females (**A**) of QurtE medaka were devoid of leucophores, and possessed fused dorsal fin and tapered anal fin, while XY males (**B**) were characterized by abundant leucophores, forked dorsal fin and fan like anal fin. *Rspo1*-OV-XY fish (**C**), despite abundant leucophores, possessed fused dorsal and tapered anal fin, and had the ability to produce viable eggs. Red and black arrowhead indicates the dorsal and anal fin, respectively.

We observed the changes in secondary sexual characteristics between normal XX, XY, and *Rspo1*-OV-XY fish under a stereomicroscope. Normal XX female medaka generally possesses short, tapering, smooth anal and dorsal fins (Fig. 2A), while XY males are characterized by long, serrated and forked fins (Fig. 2B). Additionally, the leucophores, a Y-chromosome-derived trait, which doesn’t change with phenotypic sex reversal, were used for determining the genetic sex of medaka. In contrast to normal female and male medaka, the *Rspo1*-OV-XY female possessed leucophores and female secondary sex characteristics (short anal and dorsal fins, genital papilla, egg spawning) (Fig. 2C).

**Figure 3:**
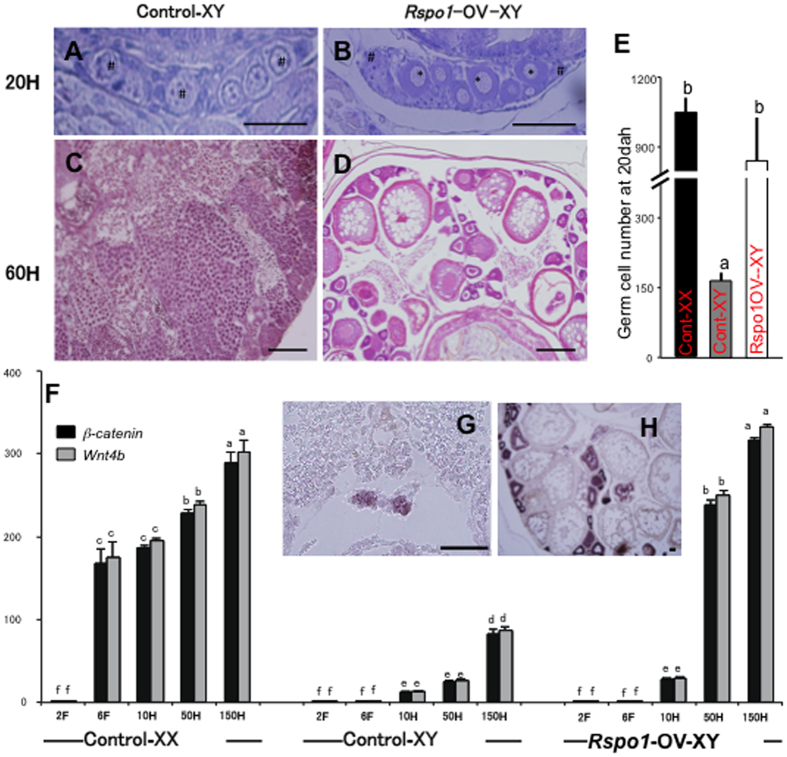
Effects of *Rspo1* overexpression on ovarian morphology and *Wnt4b/β-catenin* signaling. (**A,C**) Testis of control XY fish at 20 (**A**) and 60 (**C**) dah. (**B,D**) Ovaries of *Rspo1*-OV-XY fish at 20 (**B**) and 60 (**D**) dah. The mitotic and meiotic germ cells are marked with “*” and “^#^” respectively. (**E**) The graphical representation of total germ cells count at 20 dah in different fish groups. Excessive germ cell proliferation was observed in both control-XX (n = 10) and Rspo1-OV-XY (n = 10) gonads at 20 dah. Significant differences (marked by different letter) were detected between control-XY (n = 10) and Rspo1-OV-XY fish. (**F**) *Wnt4b* and β*-catenin* expression in control XX, control XY, and *Rspo1*-OV-XY fish at different stages of development. Marked increase in the expression of *Wnt4b* and *β-catenin* in *Rspo1*-OV-XY fish were detected at 50 and 150 dah. Data are shown as mean ± S.E., and expressed as relative abundance corrected for *Ef1α*. Different letters above the bars indicate that these groups differ significantly from each other at p < 0.01. (**G,H**) *Rspo1*-OV-XY fish shows *β-catenin* expression in small germ cell or oocytes and their surrounding cells at 6 (**G**) and 150 dah (**H**). Note: Scale bar-50 μm.

To further confirm the morphological changes in the gonads, HE (hematoxylin & eosin) staining of *Rspo1*-OV-XY and control-XY fish was performed at 20 dah and adult stages. The germ cell numbers were also counted at 20 dah to further validate the morphological observations. In medaka, slowly dividing mitotic germ cells and meiotic block define male gonadal sexuality at 20 dah. In our present study, similar characteristics were also observed in the control XY gonad (Fig. 3A,E). However, the overexpression of *Rspo1* in XY fish accelerated primordial germ cell (PGC) proliferation and preponed the meiotic initiation, before 20 dah (Fig. 3B,E). In contrast to the normal XY testis (Fig. 3C), *Rspo1* transgene subsequently activated the female developmental pathway and induced the differentiation of well-organized ovaries, including oocytes at various stages of development, as well as normal ovarian cavity in XY fish (Fig. 3D). Despite the XY genetic background, *Rspo1* overexpression resulted in complete sex reversal and production of fertile female XY fish.

### *Rspo1* affected Wnt4/β-catenin signalling

Rspo1 has been identified as an activator of Wnt4/β-catenin signalling. In order to examine the involvement of both of these genes in *Rspo1*-associated sex reversal, we measured their mRNA concentrations by real-time PCR. Our results demonstrated a female-biased expression of both of these genes throughout ontogeny. *Rspo1*-OV-XY fish showed significant induction of *Wnt4/β-catenin* after 10 dah, which eventually became non-significant compared to the control XX counterpart (Fig. 3F). *In situ* hybridization (*ISH*) analysis also confirmed the significant up-regulation of *β-catenin* in the germ cell cytoplasm of *Rspo1*-OV-XY fish at both 6 dah and adult stage (Fig. 3G,H).

### Fertility and ratio of sex reversal

To further assess the degree of *Rspo1*-associated ovarian maintenance, the gonadal morphology, papillary process, and secondary sexual characteristics of *Rspo1*-OV-XY fish were critically analysed at 90 (data not shown) and 150 dah (Fig. 2C). Furthermore, randomly selected *Rspo1*-OV-XY female fish were used to assess the mating and fertilization ability at 150 dah. Each of the transgenic fish was separately paired with non-transgenic XY fish for 3 days. Observations made within a specific time frame (30 min per pair) revealed that the sex reversed adult fish spawned daily, similar to XX female fish, and further mated with XY male fish to produce the F1 generation. Among the 38 tested *Rspo1* transgenic XY fish, nine (24%) displayed usual female-like spawning behaviour, i.e. dancing, coiling, and mating, while another 12 XY female fish (32%) did not participate in mating (SI Table 1). Subsequent gonadal histology failed to recognize any difference between these two groups. Additionally, the remaining 17 injected XY fish (44%) showed the same gonadal morphology, papillary process, secondary sexual characteristics and behaviours as normal XY fish, which might attribute to the non-functioning of Rspo1 transgene.

### *Rspo1* favoured gonadal femininity

**Figure 4:**
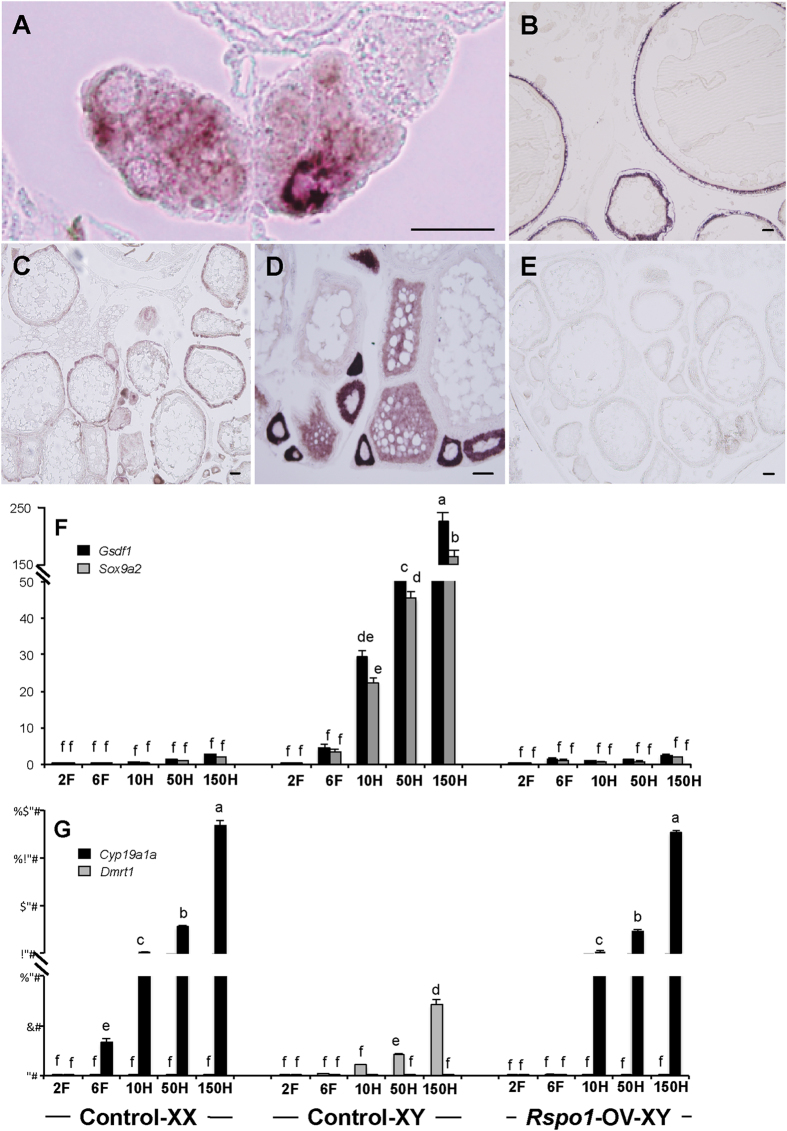
*Rspo1* overexpression modulates the expression of male and female biased genes. (**A**) Specific expression of meiotic marker (*Scp3*) were recorded in germ cells of *Rspo1*-OV-XY fish at 15 dah, suggesting the induction of female biased gonadal development in these XY fish. Strong follicular expression of *Cyp19a1* (**B**) and *Foxl2* (**C**) oocyte specific expression of *Fig1α* (**D**) and simultaneous reduction of *Gsdf* (**E**) in ovarian somatic cells were observed in adult *Rspo1*-OV-XY fish, demonstrating the female-biased gonadal development in XY fish (scale bar-50 μm). (**F**) *Gsdf* and *Sox9a2* expression in control XX, control XY, and *Rspo1*-OV-XY fish at different stages of development. Marked decrease in the expression of *Gsdf* and *Sox9a2* in *Rspo1*-OV-XY fish was observed at 50 and 150 dah. (**G**) *Cyp19a1a and Dmrt1* expression in control XX, control XY, and *Rspo1*-OV-XY fish at different stages of development. Marked increase in the expression of *Cyp19a1a* and decrease in *Dmrt1* expression in *Rspo1*-OV-XY fish was noticed at 10-150 dah. Data are shown as mean ± S.E., and expressed as relative abundance corrected for *Ef1α*. Different letters (a, b, c, etc.) above the bars indicate that these groups differ significantly from each other at p < 0.01.

To further clarify the molecular mechanism underlying *Rspo1*-induced sex reversal, real-time PCR and *ISH* were performed using male and female-dominated genes. Both these analyses showed that the representative female-specific genes (*Foxl2, Figlα, Cyp19a1a*) were markedly up regulated (Fig. 4B–D,G), while the expression of *Gsdf, Dmrt1* and *Sox9a2* (male-dominated genes) was completely repressed in the *Rspo1*-OV-XY gonads (Fig. 4E–G). Moreover, the expression of *Olvas* from 10 dah was significantly higher in *Rspo1*-OV-XY fish than its control XY counterparts (data not shown). Furthermore, the transcription of *Scp3* was detected as early as 15 dah in *Rspo1*-OV-XY fish (Fig. 4A), but absent in control XY fish.

### Involvement of *Rspo1* in the modulation of steroid production

**Figure 5:**
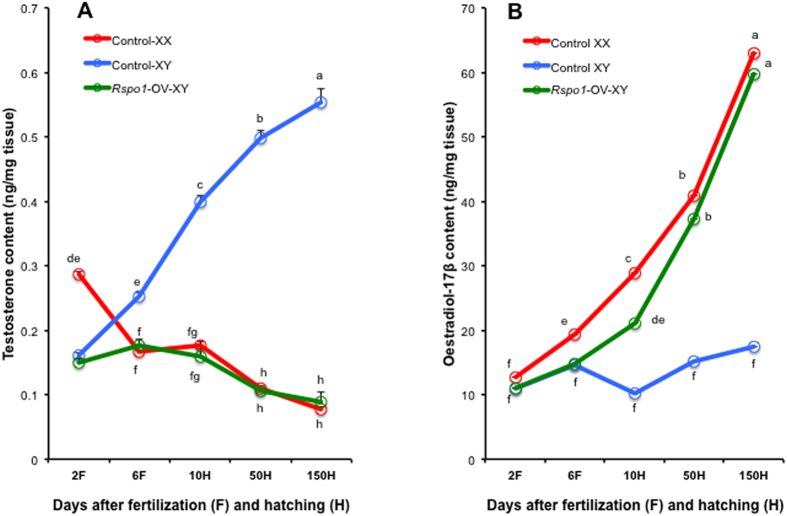
Effects of *Rspo1* overexpression on sex steroid production. Testosterone (**A**) and oestradiol-17β (**B**) levels in whole embryos and gonads in control XX, control XY, and *Rspo1*-OV-XY fish, at different stages of development. Markedly reduced testosterone and simultaneously increased estradiol-17β levels in *Rspo1*-OV-XY fish, demonstrate the female-biased steroid production in *Rspo1*-OV-XY fish. Data are shown as mean ± S.E. Different letters (a, b, c, etc.) above the bars indicate that these groups differ significantly from each other at p < 0.01.

Sex-steroid concentrations play an important role in gonadal sexuality. It was previously reported that *Cyp19b* (brain type aromatase) starts expressing from 1.5 daf in medaka^32^. Moreover, Sebillot *et al*.^33^, found that the androgen responsive spigging-1 transgene transcription starts very early in medaka (during 2-3 daf), which further suggests some possible zygotic steroid production during early stages. Similarly, in zebrafish, AR (androgen receptor) expression starts from 24 hpf (corresponds to 2 daf medaka embryos)^34^, which further strengthens the idea that some amount of steroids are being produced in fish embryos during early stages . Previously, we found that Rspo1 transcription can be affected by steroid treatment^23^ and our present data suggests that Rspo1 over expression induces *Foxl2* and *Cyp19a* expression (major genes in estrogen synthesis). So it will be critical to analyze whether Rspo1 overexpression can alter the estrogen and testosterone concentration in the body. To do so, we measured the estradiol-17β (E2) and testosterone concentrations in control XX, XY, and *Rspo1*-OV-XY F_2_ fish at different stages. No significant difference was observed in E2 production between control XX and *Rspo1*-OV-XY fish during the various stages of ontogeny (Fig. 5). Unlike control females, levels of testosterone were significantly lower in *Rspo1*-OV-XY at S-21 group samples (Fig. 5). None of the *Rspo1*-OV-XY samples showed an identical steroid profile to the control XY (Fig. 5). This prompted us to speculate whether *Rspo1* overexpression changed the characteristics of Sertoli/Leydig cells to those of granulosa/theca cells. We investigated *Cyp19a1a* (thecal cell marker) and *Dmrt1* (Sertoli cell marker) transcription in *Rspo1*-OV-XY fish at different stages (Fig. 4G), and consistent with the results for steroid production, we observed increased *Cyp19a1a* and decreased *Dmrt1* expressions in *Rspo1*-OV-XY gonads at all stages.

### Antagonism between Rspo1 and DM domain genes

**Figure 6:**
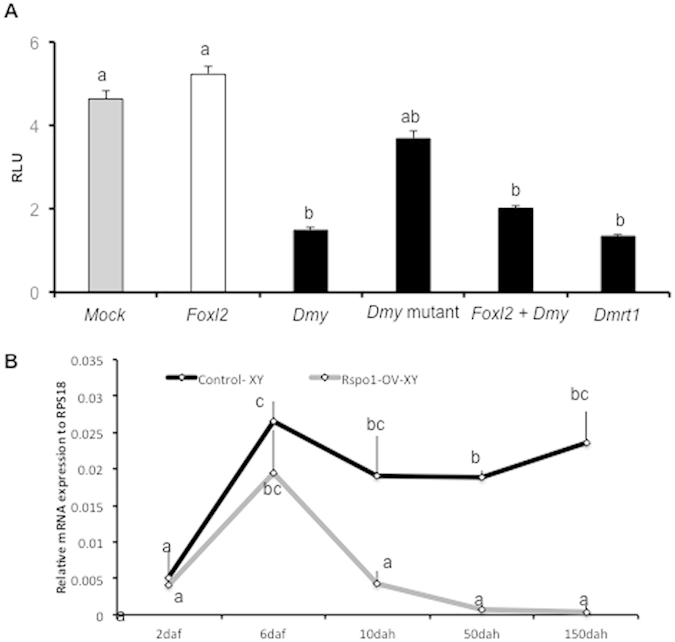
Effects of DM domain genes on *Rspo1* promoter activity. (**A**) Assessment of *Rspo1* binding potential to different male and female candidate genes. Expression plasmids of *Dmy*, mutant *Dmy, Dmrt1, and Foxl2* were co-transfected with medaka *Rspo1* (4.5 kb) promoter constructs, and relative luciferase activity (RLU) was measured after 48 hours. The RLUs of each expression plasmid (s) were plotted on Y-axis to prepare the graph. (**B**) Real-time analysis of *Dmy* expression in *Rspo1*-OV-XY fish. Marked decrease in *Dmy* expression was observed from 10 dah. Data are shown as mean ± S.E. of three independent experiments and different letters (a, b, c, etc.) above the bars indicate that these groups differ significantly from each other at p < 0.01.

*Dmy* is the first male sex-determining gene in non-mammalian vertebrates. Furthermore, *Dmrt1*, supposed to be the downstream gene of *Dmy*, located upstream of the male cascade, is essential for testicular differentiation. In contrast, *Foxl2* has been identified as a female-favouring gene in vertebrates while embryonic medaka Rspo1 expression is initiated much earlier than *Foxl2*^23^. To further investigate the transcriptional regulation of *Rspo1* gene by Foxl2, Dmy and Dmrt1, a dual luciferase promoter analysis was carried out. Our results revealed that both Dmy and Dmrt1 significantly (p < 0.01) repressed the *Rspo1* promoter activity (Fig. 6a and S2). However, no such suppression was recorded when Dmy was substituted with mutant Dmy (mutation in DM domain). In contrast to our expectations, Foxl2 failed to induce *Rspo1* transcription or reinstate Dmy inhibitory effects on the *Rspo1* promoter (Fig. 6a). To further confirm the *Rspo1* associated *Dmy* repression, we measured the expression profiles of *Dmy* by real-time PCR at different developmental stages. In the different stages of normal XY gonad, the expression of *Dmy* was very high, however, its expression was significantly repressed from 10 dah and became barely detectable by 50 and 150 dah (Fig. 6b).

## Discussion

In the present study, we demonstrated that the overexpression of *Rspo1* in XY fish induced female sex differentiation, steroid production, and sex reversal. This study showed for the first time that teleostean *Rspo1* might be sufficient to activate the canonical Wnt4/β-catenin signalling pathway and estrogen production, which, in turn, favoured femininity. We also found that DM domain genes were sufficient to antagonize *Rspo1* transcription, and vice versa, which eventually influenced the gonadal sexuality.

Since the last decade, *Rspo1* has been repeatedly proposed as one of the prime femininity regulator in the embryonic gonad^1^,^15^,^16^,^17^,^19^,^20^,^21^. In our previous study, based on an expressional analysis, we hypothesized that *Rspo1* may be critical for female sex determination/differentiation in medaka^23^. Consistent with our previous work, in the present study, we found that the overexpression of *Rspo1* induced ovarian differentiation and sex reversal in XY fish. Contrastingly, the overexpression of *Rspo1* alone in XY gonads did not result in sex-reversal in humans and mice^8^,^11^,^18^,^35^. Such anomalies may be associated with the improper activation of downstream genes/pathways. In this regard, Wnt4, an immediate downstream gene of *Rspo1*, was dose-dependently upregulated in XY sex reversed humans via the activation of Dax1 (antagonistic factor for SRY)^18^. In the present study, the *Rspo1*-OV-XY gonad also demonstrated an early surge in *Wnt4b* in the gonad, which may have escalated the chances of a gonadal sex change. It is highly likely that the overexpression of *Rspo1* induced gonadal *Wnt4b* transcription and further enhanced the production of *β-catenin* from 10 dah, reaching a similar abundance of normal XX fish by 50 dah. The stabilization of mouse *β-catenin* in the XY gonad has also been shown to induce sex reversal. In the present study, we observed an increase in germ cell-specific expression of *β-catenin* in *Rspo1*-OV-XY fish at both 6 dah and adult stages. Such tissue-specific induction could be explained using the gonad and brain-specific GFP localization data and supportive realtime profiles of *Rspo1* in *Rspo1*-OV-XY fish. Moreover, in our previous study, we showed that *Rspo1* was most abundant in the brain and gonad, the major sex-regulating organs. The considerably late, but consistent up-regulation of *β-catenin* in germ cells suggests that the elevation in *Wnt4b* and *Rspo1* or their downstream signals are sufficient to override testicular development. Hence, similar to other vertebrates, medaka *Rspo1/Wnt4*/*β-catenin* canonical signalling might play a critical role in maintaining the female sexuality.

The molecular perspective of femininity can be easily defined by the increased *Foxl2* and *Fig1a* expression in granulosa and germ cells, respectively. On the other hand, the abundant expression of *Dmrt1* and *Sox9* are indicative of gonadal masculinity. The strong expression of *Fig1a* in germ cells and *Foxl2* in follicular cells revealed that the overexpression of *Rspo1* triggers folliculogenesis and activates the female developmental pathway. Previous studies demonstrated that mouse *Foxl2* and *Rspo1* regulates distinct female sex-determining pathways and redundantly antagonizes the action of the testis determinants of *SRY/Sry*^10^,^13^,^36^. In the present study, *Foxl2* and *Cyp19a1a* (encoding the key enzyme for oestrogen production) were both up regulated in *Rspo1*-OV-XY fish. Moreover, similar to previous findings in mammalian species^10^,^13^,^36^, our promoter analysis data also suggested that *Rspo1* transcription was independent of *Foxl2*. Although both *Gsdf-Rspo1* and *Gsdf-Foxl2* co-expressing cells were prevalent in 10 dah gonads, *Foxl2-Rspo1* cells were very rare. However, at later stages (60 dah), *Foxl2* and *Rspo1* were restrictively co-localized in early germ cell surrounding cells. Therefore, most probably, the gonadal *Foxl2* surge was one of the after-effects of *Rspo1* overexpression, which helps in maintaining the femininity. We previously reported that *Dmrt1* and *Foxl2* play antagonistic roles, and the silencing of either of them, influences the gonadal sexuality in opposite directions by affecting *Cyp19a1*a transcription and oestrogen production^37^. In the present study, we also observed a significant increase in E2 production from 10 dah onwards. In medaka, *Foxl2* and *Cyp19a1a* were found to be expressed from 0 dah and 3 dah, respectively^38^,^39^. The time frame suggests that the overexpression of *Rspo1* counteracted the male gonadal development, which, in turn, induced *Foxl2* and *Cyp19a1a* transcription and further testosterone-to-oestrogen conversion.

*Dmy* and their downstream genes are known to activate the male developmental program in medaka^26^,^27^,^30^,^40^. The disruption of *Dmrt1* expression in Sertoli cells elevates *Foxl2* transcription and promotes Sertoli to granulosa cell trans-differentiation^30^, which further develops as XY female. In the present study, apart from elevated *Foxl2*, we also observed a marked reduction of DM domain genes (*Dmrt1* and *Dmy*) in *Rspo1*-OV-XY fish. Moreover, the dual luciferase promoter analysis revealed that *Dmy* and *Dmrt1* both suppressed the transcription of *Rspo1*. A ChIP assay also previously demonstrated that, *Dmy/Dmrt1b* effectively pulled down *Rspo1*^31^, which led us to hypothesize that, in normal XY males, *Dmy/Dmrt1b* suppresses *Rspo1*-activated female responsive genes and favours the male pathway. Moreover, recent studies revealed that the over-expression of *Dmy* induces testis formation in genetic XX medaka fish^29^ by suppressing female pathway genes and oestrogen production. In contrast, the overexpression of *Rspo1* in XY fish shifted the balance to the female pathway by overriding *Dmy* actions. Taking these findings into account, we speculated that the sex determination of medaka might be controlled by antagonistic actions between the female pathway (*Rspo1/β-catenin* signalling pathway) and *Dmy*-activated male pathway. A previous study showed that oestrogen treatment effectively up-regulated the expression of *Rspo1*, suppressed the *Gsdf* transcription, and induced meiosis in XY medaka^23^,^41^. Therefore, the reduction of *Gsdf* in *Rspo1*-OV-XY medaka might be attributed to excessive oestrogen production in the body. Transcriptional up-regulation of *Foxl2* in *Gsdf* expressing cells further strengthens such possibilities of enhanced oestrogen production. Unlike mammals^16^,^17^, our result suggests that *Rspo1* possesses the potential to activate female-dominated gene transcription in both somatic and germ cells^23^ by antagonizing the actions of male pathway genes (*Dmy, Gsdf, etc.*). Thus, this unique expression profile is presumably an important factor for successful sex reversal.

Proliferative mitosis and meiotic initiation are considered to be the first phenotypic sign of female sex differentiation^25^,^42^. The overexpression of *Rspo1* in XY fish accelerated the mitotic burst and meiotic initiation of germ cells at 15 dah. A previous study demonstrated that the expression of *Dmy* activates the male developmental pathway by suppressing the proliferation of PGC and meiotic initiation in XY male medaka^28^,^29^. In our experimental fish, meiotic germ cell division was observed at 0 dah in XX ovary, while meiotic germ cells could not be detected until 40 dah in XY testis. The excessive germ cell proliferation and expression of *Scp3* (meiotic marker) in *Rspo1*-OV-XY fish, at 15 dah, implied that the *Rspo1* transgene preponed the timing of meiosis in XY fish. Unlike normal XX medaka juveniles, no sign of proliferative mitosis or meiosis was observed in *Rspo1*-OV-XY fish until 5 dah. We hypothesized that the delayed meiosis in Rspo1-OV-XY fish might be due to the postponed up-regulation of female-biased genes (*Wnt4, β-catenin* and *cyp19a1a*) and oestrogen production around 10 dah. Available reports suggest that, in embryonic medaka gonads, *Dmy* and *Gsdf* are able to repress germ cell proliferation and meiotic initiation^28^,^29^. Similarly we also observed significant down-regulation of male-biased genes at 10 dah *Rspo1-*OV-XY fish. This, together with the suppressed *Dmy/Gsdf/Sox9a2* pathway, indicates that *Rspo1* favours gonadal trans-differentiation, by antagonizing the functions of downstream genes in the male pathway. several researchers have demonstrated that androgens are the key regulator of male secondary sex development^43^,^44^. In the present study, the overexpression of *Rspo1* changed the secondary sex characteristics and induced the development of female-like dorsal and anal fins. The sex-reversed XY female fish possessed both leucophore and female-specific secondary sex features (short anal and dorsal fins, round genital papilla, egg-spawning ability). This strongly suggested that the overexpression of *Rspo1* resulted in a decrease in the production of androgens, as revealed by the reduced transcription of *Cyp11b* (key enzyme for 11-KT biosynthesis) in XY fish. In accordance, testosterone concentrations were markedly reduced in *Rspo1*-OV-XY fish. This reduction might be associated with depleted number of testosterone-producing cells or increased oestrogen production^38^.

Taken together, our results indicate that *Rspo1* plays a critical role in ovarian differentiation by antagonizing the male axis and activating female-specific genes in the medaka gonad. In females, although *Rspo1*-activated signalling and *Foxl2*-responsive E2 production pathways were mutually exclusive, they acted synergistically to promote the female pathway. Therefore, considering the fact that *Rspo1* is one of the first female biased gene expressed in medaka^23^, we postulate that *Rspo1* is a foremost factor that shifts the balance towards femininity. In medaka, the balance between *Dmy* and *Rspo1* transcription determines the fate of the gonad. The activation of *Dmy* induces male differentiation, while *Rspo1* favours femininity. Our results further demonstrate that oestrogen-producing and *Rspo1*-activated-signaling pathways, although independent, mutually ensure the female sex determination/differentiation in fish. In summary, our finding suggests that ectopic *Rspo1* expression in XY medaka sufficiently induces ovarian differentiation by suppressing the master male sex determining gene (*Dmy*) and other male biased genes, elevating ovarian specific gene expression, and altering the steroid production. To our knowledge, the present work is the first report in vertebrates highlighting that Rspo1 alone can completely overturn the genetic male cascade to induce female development. However, further studies are essential to clarify the *Rspo1* associated *Dmy* suppression mechanism in medaka.

## Methods

### Fish strain and husbandry

The QurtE strain of medaka was used in this study. This strain expresses a male-specific leucophore that facilitates the easy sexing of the fish. Genetic sexing was performed using previously reported protocols^26^. All fish were maintained at 26 ± 2 °C under a 14-h light and 10-h dark cycle. Eggs were collected within 30 min of fertilization and incubated in distilled water (milli-Q) containing an antifungal solution (Methylene blue, 0.0001%) at 26 ± 2 °C. Brooders and juveniles were fed fresh artemia, while larvae were given artificial food. All *in vivo* experiments and fish maintenance were conducted following protocols and procedures approved by the Institutional Animal Care and Use committee at Ehime University, Japan, and Southwest University, China.

### Plasmid construction

The total RNA of adult ovaries was extracted using the RNeasy mini kit (Qiagen, USA) and cDNA was subsequently synthesized using the Omniscript kit (Qiagen). The ORF of *Rspo1* was amplified from the adult ovaries, using two adapter-tagged gene-specific primers (SI Table 2), digested with *Sac* II and *BamH* I (New England Biolabs, UK), purified and cloned into similarly digested pIRES-hrGFP-1a vector (Stratagene, USA).

Genomic DNA was isolated from the caudal fin of adult fish using the DNeasy Blood and Tissue kit (Qiagen). The medaka *Rspo1* promoter region (−4449bp) was PCR amplified (SI Table 2), cut with restriction enzymes (*Mlu* I and *Xho* I), and then directionally inserted into the pGL3-basic vector (Promega, USA). The transcription factors of *Foxl2, Dmy* and *Dmrt1*, were sub-cloned into pcDNA3.1 (Invitrogen, USA) from the original clones using gene-specific ORF primers. The inserts and directions of all the plasmids were confirmed by subsequent sequencing. The constructs with correct inserts were purified using the QIAfilter Plasmid Midi kit (Qiagen) and used for subsequent microinjections and luciferase assay.

***FISH***. The 10 and 60 dah Rspo1-OV-XY medaka gonads were fixed overnight in 4% paraformaldehyde (Nacalai tesque, Kyoto, Japan) at 4 °C. After fixation, the gonads were embedded in paraffin and sectioned at 30 μm, and subjected to *FISH*. To further confirm the cellular localization of GFP signals in the gonad of *Rspo1*-OV-XY fish, expression of *Gsdf* (Sertoli cell), *Rspo1* and *Folx2* (follicular cell) were investigated at 10 dah, meanwhile, *olvas* (germ cell), *Rspo1* and *Foxl2* expression were also examined at 60 dah. *FISH* was performed as described previously^23^. Briefly, probes were labeled with fluorescein isothiocyanate (FITC), Digoxigenin (DIG) and Biotin (Roche, Germany), and Alexa fluro-488-anti-FITC, Alexa fluro-546-anti-DIG, and Alexa fluro-591-anti-biotin antibodies were used for the detection. Nuclear staining was carried out using Hoechst dye according to the manufacturer’s instruction (Thermo Scientific, USA). Signals were observed and photographed by a confocal microscope (Zeiss 710, Carl-Zeiss Germany).

### Microinjection and detection of transgenes

Microinjection was performed according to the previously reported protocol^26^. Briefly, fertilized eggs were collected within 15 min of spawning, cleaned off the attaching filaments and injected with linearized *Rspo1*-GFP plasmid DNA (50 ng/μl in Yamamoto’s solution). Embryonic *Rspo1*-GFP expression was traced under the microscope at different developmental stages. Genomic DNA was isolated from tail clippings and genetic sex was confirmed by PCR using gene-specific primers (SI Table 2). GFP-expressing genetic XY individuals were sacrificed at 20, 60, and 150 dah to analyse the effects of *Rspo1* overexpression by real-time PCR, H&E (Haematoxylin and Eosin) staining, and *ISH*. The remaining GFP-positive XY fish were further reared until adult stage to assess the sex reversal status, secondary sexuality, breeding ability, and fertilization potential. Furthermore, sex-reversed XY-females were crossed with normal males to determine fertility and produce F_1_ and F_2_ generation progeny. Genomic DNA was extracted from the tail of XX/XY control and XY transgene fish of the F_0_ and F_1_ generations using the DNeasy Blood and Tissue kit (Qiagen), according to the manufacturer’s protocol. The transgene integration status was examined using a gene-specific forward primer and vector-based reverse primer (SI Table 2). Integration PCR was carried out as follows: 5 min at 94 °C, followed by 40 cycles of 15 sec at 96 °C, 1 min at 68 °C, and a final step of 7 min at 72 °C. The amplified fragment was sub-cloned into pGEM-T easy vector and confirmed by sequencing.

### Histology and ISH

At 20 and 60 dah, the trunk portions of control XX, control XY, and *Rspo1* transgene XY fish were fixed in Bouin’s fixative, embedded in paraffin, sectioned at 5 μm, and subjected to standard HE staining. 10 individual gonads of 20 dah group samples were serially sectioned (5 μm each), HE stained and total germ cell count was performed using stereo-microscope (400X zoom). The average of 10 individuals was used for graphical representation. Samples (6, 15 and 60 dah), fixed in paraformaldehyde (4%), were embedded in paraffin and sectioned at 5 μm for *ISH. ISH* was carried out using sense and anti-sense digoxigenin-labelled RNA probes transcribed *in vitro* with an RNA labelling kit (Roche) from their respective plasmid DNAs containing the ORFs of medaka *Foxl2, β-catenin, Figlα, Gsdf, Scp3,* and *Cyp19a1a*. Sections for *ISH* were deparaffinised, hydrated, and treated with proteinase K (10 μg/ml, Roche), and then hybridized with the sense or anti-sense DIG-labelled RNA probe at 58 °C for 22 h. Hybridization signals were then detected using alkaline phosphatase-conjugated anti-DIG antibody (Roche, Germany) and NBT, as described previously^45^.

### Quantitative gene expression

Changes in gene expression were quantified using the ABI Prism 7000 sequence detection system (Applied Biosystem, USA). One hundred nanograms of total RNA, isolated from embryos or gonads at different developmental stages (2 and 6 daf, 10, 50, and150 dah) were used for cDNA synthesis, using a Quantitect RT PCR kit (Qiagen). First strand cDNAs were diluted to 100 μl for subsequent use. Gene-specific RT-PCR was performed using the SYBR green master mix (Applied Biosystem) and 5 ng of cDNA, according to the manufacturer’s instructions. Real-time PCR primers are listed in SI Table 2. PCR conditions included an initial denaturation at 94 °C (2 min) followed by 40 cycles at 94 °C (30 s) and 60 °C (1 min). *Ef1α* was used as the internal control. The absolute transcript copy number of each gene was determined with the help of appropriate standard curves and normalized with the *Ef1α* copy numbers in each sample. The reported values were averaged from sample triplicates of three pooled whole body samples (10 individuals from 2 and 6 daf, and 10 dah, and three gonads from 60 and 150 dah) of XX, XY and *Rspo1*-OV-XY. The RNAs were used for two independent cDNA synthesis, each group of cDNA were further analysed twice and all the data were used for mean and standard error calculations.

### Steroids measurement

One hundred de-yolked embryos (2 and 6 daf)/juveniles (10 and 50 dah)/10 adults (150 dah) were quickly frozen in liquid nitrogen, weighed, homogenized, and used for steroid extraction. The freeze-dried extracts were diluted in appropriate quantities of dilution buffer (20 μl per mg tissue) and used to measure testosterone concentrations with the testosterone high sensitivity ELISA kit (Enzo, Japan), following manufacturer’s instructions. Samples were further diluted (100-fold) to measure oestrogen with the estradiol-17β high sensitivity ELISA kit (Enzo). Steroid concentrations were calculated based on the standard curve prepared using respective steroids, provided by the manufacturer (Enzo). Both tissue and extract duplicates were analysed before final calculations. Preliminary analyses were carried out to standardize the sampling method and assessment protocols.

### Luciferase assays and repression of *Dmy* by Rspo1 transgene

HEK293 cells were grown in DMEM (Sigma) supplemented with 10% foetal bovine serum (JRH Biosciences) and 1Χ penicillin-streptomycin-glutamine (Invitrogen) with 5% CO_2_ at 37 °C. Confluent HEK293 cells were seeded on 24-well plates (at 5 × 10^5^ cells/well), grown for 24 hours, and transfected using Lipofectamine (Invitrogen) with the following plasmids: 1) 0.5 μg of constructs of the *Rspo1* promoter cloned into the pGL3-basic luciferase reporter plasmids; 2) 0.05 μg–0.5 μg of the pcDNA3.1 expression plasmids (Invitrogen) of *Dmy* and *Dmrt1*, and 3) pRL-TK (Promega), 100 ng/well. Renilla luciferase was employed as an internal control for transfection efficiency. The transfection solution was made of 100 μl of Opti-MEM I reduced serum medium containing complexed DNA, and 2 μl of Lipofectamine reagent. After 48 h of transfection, the cells were PBS-washed and lysed with 100 μl luciferase lysis buffer. Luciferase activity was measured using the Dual-Luciferase Reporter Assay kit (Promega) and LUMATLB 9507 luminometer (Berthold Technologies GmbH & Co. KG). Relative luciferase activity was calculated by dividing firefly luciferase activity by Renilla luciferase activity. Results are presented as the means ± S.E. of triplicates. Moreover, the expression level of *Dmy* was compared between the normal XY and *Rspo1*-OV-XY fish by real-time PCR, according to the methods aforementioned. The copy number of *Dmy* was measured at 2 and 6 daf, 10, 50 and 150 dah, and data were expressed as the relative mean copy number ± S.E. at each stage.

### Statistical analysis

Statistical differences in relative mRNA expression, steroid concentrations, and relative luciferase activity were assessed by one-way ANOVA, followed by Tukey’s test or the Student’s *t*-test. All statistical analyses were performed using GraphPad prism software. All experimental data are shown as the mean ± S.E. Differences were considered significant at p < 0.01, if not otherwise stated.

## Additional Information

**How to cite this article**: Zhou, L. *et al*. Rspo1-activated signalling molecules are sufficient to induce ovarian differentiation in XY medaka (*Oryzias latipes*). *Sci. Rep.* **6**, 19543; doi: 10.1038/srep19543 (2016).

## Supplementary Material

Supplementary Information
