# Supplementary material for: Rspo1-activated signalling molecules are sufficient to induce ovarian differentiation in XY medaka (Oryzias latipes)
Source: Sci Rep. 2016 Jan 19;6:19543. doi: 10.1038/srep19543 (PMC4726049; doi:10.1038/srep19543)
Supplement: Supplementary Information [file srep19543-s1.pdf]

Rspo1-activated signalling molecules are sufficient to induce ovarian differentiation in XY medaka (*Oryzias latipes*)

Linyan Zhou[1, 2, #], Tapas Charkraborty[2, 3, #], Qian Zhou[4, #], Sipra Mohapatra[3], Yoshitaka Nagahama[2, 3, 5, *] and Yueguang Zhang[1, *]

Supplementary figures

Figure S1. **Transgene construction and assessment of transgenesis**. (A) Schematics of pIRES-hrGFP1a-Rspo1 overexpression plasmid. (B) Genotyping and integration check of *Rspo1*-overexpressed adults. Upper panel, abundant amplifications were detected in both F_0 and F_1 (XY fish) injected with pIRES-hrGFP1a-Rspo1, but no amplification was found in control XX and XY fish. Lower panel, genetic sexing of control and transgenic medaka by genomic PCR, where 2 bands (Dmy and Dmrt1) indicates genetic male, while 1 band (Dmrt1) represents female. (C) Analysis of tissue specificity of hCMV promoter driven *Rspo1* transgene in F_0 generation. Real-time *Rspo1* mRNA expressions were highly localized in gonads and brain, while significantly low expressions were recorded in the remaining body (RB). Data are shown as mean ± S.E. of three independent experiments and the letters (a, b, c, etc.) above the bars indicate that these groups differ significantly from each other at $p<0.05$.

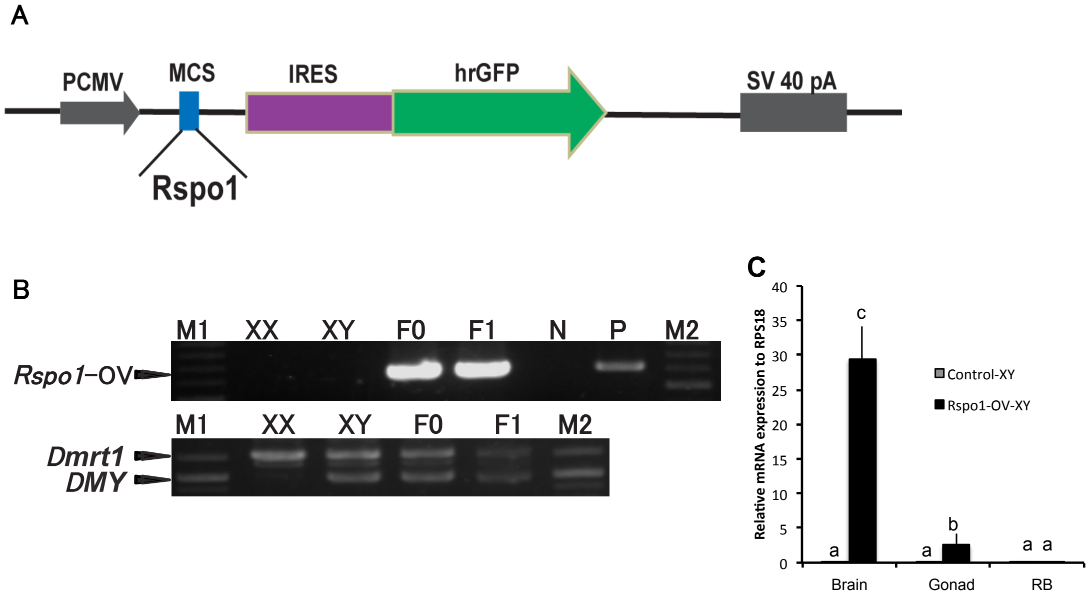

Figure S2. **Minimal promoter analysis of *Rspo1***. *Dmy* expression plasmid were co-transfected with different sized medaka *Rspo1* (1-6 kb) promoter constructs, and relative luciferase activity (RLU) was measured after 48 hours. The RLUs of each expression plasmid (s) were plotted on Y-axis to prepare the graph. Data are shown as mean ± S.E. of three independent experiments and the letters (a, b, c, etc.) above the bars indicate that these groups differ significantly from each other at $p<0.01$. Note: the length of different promoter constructs were determined empirically using the availability of potential *DM* domain binding sites.

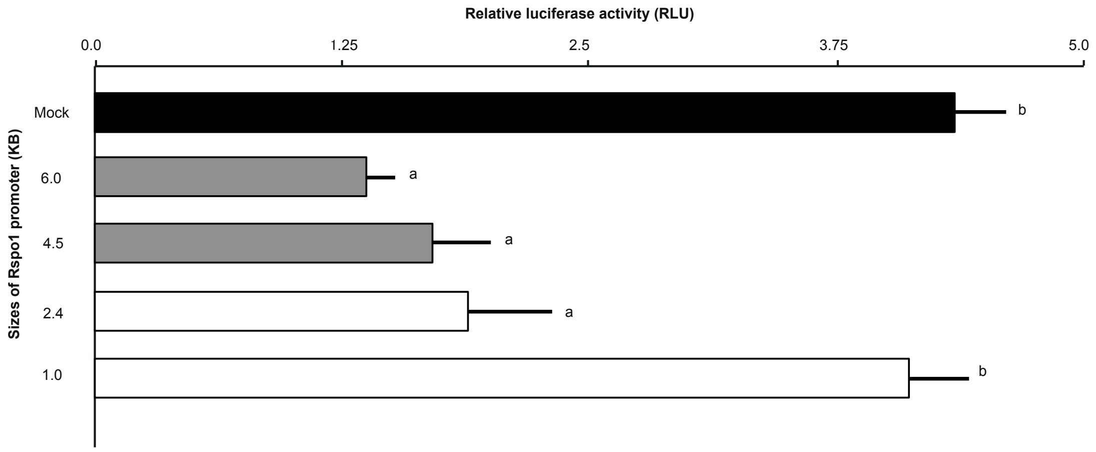

Supplementary table

SI Table 1: Screening of Rspo1-OV-XY F_0 progenies.

Genotype	Phenotype		Fertility		
XY	female	male	spawning	non-spawning	spermiation
**38**	**21**	**17**	**9 (24%)**	**12 (32%)**	**17 (44%)**

SI Table 2: List of major primers used in this study.

Primer name	Primer sequence (5' to 3')	Purpose
Rspo1-GFP-F	GAGCTCCCGCGGATGCAGCTGGGACTGGTG	Amplification
Rspo1-GFP-R	GGATCCGAATTCGGTGACGGAGCTGGTTGTG	of Rspo1
Rspo1-gPCR-F	ACATGTGGGTTTAAGAAAGGC	Genomic PCR
Rspo1-gPCR-R	GTCCTTATCATCGTCGTCTT	*Rspo1* transgene
DMY-gPCR-F	CCG GGTGCCCAAGTGCTCCCGCTG	Genomic PCR
DMY-gPCR-R	GATCGTCCCTCCACAGAGAAGAGA	of *DMY*
β-catenin-qPCR-F	ACTGGATATCGGAGCACAGG	Real-time PCR
β-catenin-qPCR-R	CAGGGAGAGCATCAGTGTGA	
Dmrt1-qPCR-F	TCCTCCTACTATGGAAACCTGTACCA	
Dmrt1-qPCR-R	GAAGGAGTGCATGCGGTACTG	
Cyp19a1a-qPCR-F	AGCTTATTTTGCCCAAGGCC	
Cyp19a1a-qPCR-R	TTGAGCAGCAGGAGCATGAAA	
Gsdf-qPCR-F	GGGCTGGACACTATTCGAGA	
Gsdf-qPCR-R	CATGACACAGAGGAGCTGGA	
Dmy-qPCR-F	TCCTATTATGGAAACCTGCACAACTAC	
Dmy-qPCR-R	GAAGGAGTGCATGCGGTACG	
Sox9-qPCR-F	CTCCGACGCTCCCAGTCCCA	
Sox9-qPCR-R	GACGGCCGGGTGTTTTCGGT	
Wnt4b-qPCR-F	CTGGAAAGTCATGCCTCCAT	
Wnt4b-qPCR-R	TACGCGTTAAGCGAACCTCT	
EF1 α -F14	CAGCTTCAACGCTCAGGTCAT	
EF1 α -R14	TGAACTTGCAGGCGATGTGA	
Rspo1-pro-F	GGGTACCACGCGTGCCTGCCCCTTATGGAGCAG TAATACTACC	Amplification of promoter
Rspo1-pro-R	GAGATCTCTCGAGGATGATCCCGCTTTTCTCCTG	region
